# The Influence of Hypothermia Hibernation Combined with CO_2_ Anesthesia on Life and Storage Quality of Large Yellow Croaker (*Pseudosciaena crocea*)

**DOI:** 10.3390/foods11040514

**Published:** 2022-02-11

**Authors:** Nanfeng Tan, Yuanpei Gao, Yueke Wang, Shanggui Deng, Pengxiang Yuan, Tong Jiang, Wanyuan Zheng

**Affiliations:** 1 Key Laboratory of Health Risk Factors for Seafood of Zhejiang Province, College of Food Science and Pharmacy, Zhejiang Ocean University, Zhoushan 316022, China; 15013827529@163.com (N.T.); gaoyuanpei89@163.com (Y.G.); wangyueke0128@163.com (Y.W.); dengshanggui@163.com (S.D.); 2 College of Biosystems and Engineering and Food Science, Zhejiang University, Hangzhou 310000, China; 3 National Institute for Nutrition and Health, Chinese Center for Disease Control and Prevention, Beijing 100000, China; jiangtong@ninh.chinacdc.cn; 4 Zhejiang Industrial Group Co., Ltd., Zhoushan 316000, China; zwy0960@163.com

**Keywords:** fish, live transportation, hypothermia hibernation, CO_2_ anesthesia, preservation

## Abstract

We explore the feasibility of the long-term transportation of live large yellow croakers (*Pseudosciaena crocea*) using the combined method of CO_2_ anesthesia and hypothermia hibernation, and its effect on the quality of recovered fish stored at 4 °C. Fish treated with CO_2_ anesthesia at a 2 ppm/s aeration rate were cooled at 3 °C/h to hibernate survived for 36 h at 8 °C in seawater. This method resulted in better survival rates and time, and a lower operational time than hypothermia hibernation or CO_2_ anesthesia methods. The results of a blood analysis indicated that the stress experienced by the fish during hibernation was mitigated, but existent after recovery. The drip loss rate of the ordinary muscle of hibernated fish was significantly different from that of the control group at 4 °C, but there was no significant difference in the pH, lactic acid content, and color during early storage. Furthermore, hibernation did not affect springiness and chewiness. Thus, the combination of CO_2_ anesthesia and hibernation may improve the survival and operation efficiency of fish in long-term transportation. However, this method affects the quality of fish after long-term storage. Thus, hibernated fish should be consumed after appropriate domestication or immediately after recovery.

## 1. Introduction

Live marine fish are becoming more popular in the global market; thus, improving their transportation or preservation techniques is necessary. The large yellow croaker (*Pseudosciaena crocea*), is an important and economically valuable fish in the world, especially in the East China Sea [1]. However, large yellow croakers die easily during live transportation owing to their timidity, resulting in extreme stress responses to manual operation, noise, and transportation, even in an oxygen-rich environment [2]. Thus, they are often transported to market in a chilled or frozen state. Although live large yellow croakers can retain maximum flavor and nutritional value, their edible value and economic benefits are reduced when chilled and frozen [3]. Thus, there are a lot of possibilities for developing a live transport method of the large yellow croaker. There are few studies on the live transportation of the large yellow croaker, which remains the limiting factor in their successful mass marketing.

Hibernation is generally considered a useful method for improving the survival rate of fish by reducing the respiration, metabolism, and stress responses during handling, transport, and other manipulations in aquaculture [4]. Poikilotherms, such as fish, go into hibernation due to the gradient cooling of the environmental temperature to its hibernation temperature zone. For example, the crucian carp can hibernate for 38.0 h at a low temperature (8.0 °C) [5]. However, rapid cooling can automatically activate defense mechanisms, which cause the dysfunction and death of fish cells [6]. Bai et al. [7] indicated that channel catfish subjected to increasing chilling rates showed lower survival rates and shorter preservation times; its survival rate after preservation for 15.0 h decreased from 80.0% at a cooling rate of 2.0 °C/h to 40.0% at 4.0 °C/h, and 0.0% at 6.0 °C/h. Therefore, to be effective, the hibernation technique adopted needs to be time-consuming and involve a very slow cooling method to reduce the variable temperature stress of fish.

Moreover, anesthesia is important whenever fish are handled to reduce handling stress and mortality. Anesthesia combined with hibernation has been shown to improve survival rates. Hypothermia combined with benzocaine effectively reduced scale loss during the transportation of juvenile *Chirostoma estor* [8]. Artificial hibernation by the combination of eugenol and low temperatures proved to be an effective method in the transportation of live crucian carp, which did not induce cellular damage [5]. However, the use of anesthetics, such as the aforementioned benzocaine and eugenol, in edible fish remains controversial because of the adherence to the drug regression period before the fish is eaten [9]. Alternatively, carbon dioxide (CO_2_) dissolved in water is an effective anesthetic in various aquatic animals [10], and is ideal for creatures intended for human consumption, since no toxic substances remain in the organisms [11,12]. However, CO_2_ is only suitable for short-term anesthesia, and prolonged CO_2_ anesthesia is likely to induce death due to respiratory failure [13,14]. The development of a new green method utilizing hypothermia hibernation with CO_2_ rapid hypnosis for the long-term transportation of marine fish is worth investigating using the live large yellow croaker as a model.

In addition, the physicochemical properties of muscle are affected by stress during fish transport [15]. For this reason, Sensory evaluation of fish muscle including color, texture, appearance, odor, etc. is a parameter of utmost importance for the appraisal and selection of fish by consumers. Storing live fish in a low temperature and CO_2_ anesthetic conditions would likely affect their quality; thus, quality changes in fish muscles after hibernation should also be considered. However, there are few reports on the effects of hibernation on fish quality during storage.

In this study, the feasibility of long-term transportation of large yellow croakers using a combination of CO_2_ anesthesia and hypothermic hibernation is determined. Furthermore, the effect of hibernation on fish quality compared with that of fish chilled at 4.0 °C is explored.

## 2. Materials and Methods

### 2.1. Study Samples

Cultured (mariculture) large yellow croakers (503.47 ± 25.38 g and 32.71 ± 1.03 cm in length) were obtained from Zhejiang Zhoushan Peninsula Aquaculture Co., Ltd. The average seawater salinity in the aquaculture area (the East China Sea) was 22.0‰ and the pH value was 7.2. The large yellow croakers used in the experiment were healthy, disease-free, and had a good growth trend; the fish were transported alive to the laboratory and individually cultured in a 10.0 L seawater tank at 15.0 °C with continuous aeration. The process of transport and transfer needed to be fast, but gentle enough to minimize the stress of the fish. Even so, the fish were cultured for 2–3 h before the experiment to ensure their condition was stable.

The hibernated and nonhibernated (hereafter referred to as controls) large yellow croakers were killed by swiftly cutting through their hindbrains for preservation experiments; each side of the fish was immediately cut off in fillets (1.5 cm thick) perpendicular to the direction of the body length. The fillets of the hibernated fish and controls were kept in polyethylene bags and stored at 4.0 °C, and the change rate of drip loss, pH, lactic acid content, color, and physical texture (springiness, chewiness) of muscle were measured to study the effects of hibernation on fish quality.

### 2.2. Anesthesia and Hibernation Conditions of Large Yellow Croaker

#### 2.2.1. Trial 1, CO_2_ Anesthesia

Large yellow croaker fish demonstrate strong stress responses and high mortality rates under continuous aerated CO_2_ [16,17]. Here, into the seawater, CO_2_ was intermittently aerated for 10.0 s at 1.0 min intervals from a CO_2_ cylinder through a plastic hose and air stone. An MF-5706 gas flow meter (Siargo company, Suzhou, China) was used to control the rate of aeration. The aeration process was repeated at different rates (2.0, 7.0, 12.0, 17.0, and 22.0 ppm/s) until the large yellow croaker entered the anesthesia state, which was denoted by a rollover and loss of reactivity to external stimuli [18]. The anesthetized fish were moved into seawater at 15.0 °C to recover after certain time intervals. The CO_2_ aeration time, as well as the survival times and rates of the fish at different aeration rates were recorded.

#### 2.2.2. Trial 2, Hypothermia Hibernation

Our preliminary experiments found that large yellow croakers rolled over, struggled, and exhibited slowed breathing and reduced sensitivity to external stimuli when the temperature was dropped to 8.0 °C. Furthermore, we observed that the nasal cavity was the easiest avenue to freeze (−1.3 °C) various tissue or organs of large yellow croakers. Theoretically, the hibernation temperature zone of the large yellow croaker ranges from −1.3 to 8.0 °C. Other live large yellow croakers, as well as those stored in the tank (15.0 °C) with continuous aeration, were placed in a refrigerator and the seawater temperature was dropped to 8.0 °C at different cooling rates (1.0, 3.0, 5.0, 7.0, and 9.0 °C/h). After the fish were hibernated, the temperature of seawater was kept at 8 °C. The hibernated fish were moved into seawater at 15.0 °C to recover after certain time intervals, and the effect of cooling rates on fish survival rate was investigated.

#### 2.2.3. Trial 3, the Combined Storage Method of CO_2_ Anesthesia and Hypothermia Hibernation

Another batch of live large yellow croakers was first treated by the optimal CO_2_ anesthesia condition, determined in the preceding experiment, in a 10.0 L seawater tank (15.0 °C). The anesthetized fish were immediately transferred to another 10.0 L seawater tank (15.0 °C) with continuous aeration, and the tank was then put into the refrigerator, where the temperature of seawater was lowered to and maintained at 8.0 °C at different cooling rates (1.0, 3.0, and 5.0 °C/h). The hibernated fish were moved into seawater at 15.0 °C to recover after certain time intervals. The survival rate and survival and recovery times were measured, and the feasibility of the combined long-term hibernation method of large yellow croakers was determined. The hibernation and control groups used to study the effects of hibernation on fish quality during storage were killed immediately after recovery and stored until ready for further analysis.

### 2.3. Determination of Blood Components, Glucose Levels, and Total Serum Protein of the Large Yellow Croaker Using the Optimal Combined Method

Additionally, blood components, blood glucose, and total serum protein of the fish treated by the optimal combined method were measured before and during hibernation and after recovery to study the effects of hibernation. The blood was collected from the caudal vasculature of the live fish using a 5.0-mL vacuum tube with anticoagulant (Tianai Medical Instrument Co., Ltd. Qingdao, China) before and during hibernation and after recovery. The blood tests were conducted in Meikang Biotechnology Co. Hangzhou, China using a Hitachi 3100 automatic biochemical analyzer.

### 2.4. Determination of Drip Loss

Drip loss of the fillets of hibernated and nonhibernated fish was measured according to He et al. [19]. The fresh fillets were weighed (W_1_), wrapped, and suspended in a polyethylene bag, and stored at 4.0 °C; the fillets were then re-measured (W_2_) after certain time intervals. The reference value of drip loss of each sample (W) was expressed as a percentage of weight loss and calculated using the following formula: W = (W_1_ − W_2_)/W_1_.

### 2.5. Determination of pH and Lactic Acid

Since pH values can vary between heat and cold-stressed red and white muscle in fish [20], changes in the muscle pH were measured only for the dorsal ordinary muscle (white muscle) of hibernated and nonhibernated fish, using an AZ8695 pen touch pH meter (Taizhou Taixin 153 Technology Co., Ltd., Taizhou, China). Each fillet was measured five times and the average value was used as the final value. The lactic acid content of the muscle was also determined using a lactic acid (LD) test kit (Nanjing Jiancheng Bioengineering Institute, Nanjing, China) according to the manufacturer’s instructions.

### 2.6. Determination of Color Variations

Color index in the ordinary dorsal muscle was closely associated with the content and chemical state of hemoglobin, which mainly caused changes in a* and L* [21,22]. The color variations in a* and L* of dorsal ordinary muscle was measured using a CM-5 chromameter (KONICA MINOLTA, Japan) [23]. a* and L* signified the color variations of green to red chromaticity and black to white chromaticity, respectively.

### 2.7. Determination of Texture

The springiness and chewiness of the dorsal ordinary muscle (white muscle) of the fish fillets were measured using the TMS-PRO physical property analyzer (FTC company, Sterling, VA, USA) with a flat-bottom cylindrical probe (diameter 5.0 mm). The starting force, measuring force, shape variable, and probe speed were set as 0.6 N, 60.0 N, 30.0%, and 60.0 mm/min, respectively.

### 2.8. Statistical Analysis

To reduce the individual differences among the large yellow croakers used in the study, some data were expressed as the rate of change. Data were expressed as mean values ± standard deviation (SD) for each group. SPSS software (v 21.0) was used for all the data analysis. Differences among the multiple treatment and control groups were determined using a one-way analysis of variance, followed by the least significant difference post hoc test. A probability level of *p* < 0.05 was considered to denote statistical significance.

## 3. Results and Discussion

### 3.1. The Effects of Anesthesia and Hibernation on the Live Large Yellow Croaker

When only CO_2_ (aeration rate: 2.0, 7.0, 12.0, 17.0, and 22.0 ppm/s) was used to anesthetize the large yellow croakers, the fish struggled, initially displaying shortness of breath, followed by a loss of equilibrium or turning over of the body. These responses reduced the sensitivity to external stimuli. Each group was anesthetized and intermittently aerated with CO_2_ three times. After the hibernated fish were transferred to seawater at 15.0 °C, the fish took an average of 10.5 ± 0.6 min to resume an upright equilibrium and normal swimming motion. Their survival rate and time decreased as the aeration rate increased (Table 1), as 90.90 % of the fish could live for 3.5 h when the aeration rate was 2.0 ppm/s. This rapidly decreased to 58.33% for 2.5 h when the aeration rate increased to 7.0 ppm/s. This may be because the rapid increase in CO_2_ concentrations inhibited the loading of oxygen on red blood cells, resulting in hypoxic respiratory failure [10,14]. Our results suggested that the aeration rate of CO_2_ at 2.0 ppm/s was the optimal anesthesia condition for the large yellow croaker.

The survival rate and times also decreased as the cooling rate increased (Table 1), 90.00% of the fish could live for 30.0 h when the water was cooled at 1.0 °C/h, and this decreased to 54.54% for 26.0 h when the cooling rate was increased to 3.0 °C/h. The recovered fish took an average of 15.2 ± 3.5 min to resume an upright equilibrium and swimming motion. Their survival rates and times were similar to that of the channel catfish reported by Bai et al. [7], who explained that an acute temperature change might lead to cellular dysfunction in fish, whereas the appropriate gradual change in temperature preserved ion transport regulation via the adaptation of the plasma membrane lipids. In addition, the stress caused by the cold may motivate the mRNA expression of apoptosis-related genes, which inhibit the protective mechanisms against the temperature and the cooling rate reaching a critical level, thereby leading to damage to cellular components and elevated levels of apoptotic cell death [24]. Thus, the cellular structure and function of the fish could be maintained over a defined range of gradual temperature changes as rapid changes in ambient temperature may cause strong stress responses in the fish leading to an increase in mortality.

Nevertheless, the cooling rate of 1.0 °C/h required 7.0 h to drop from 15.0 °C to the highest critical hibernation temperature (8.0 °C), meaning the hypnotic efficiency was very low. Importantly, the CO_2_ aeration rate at 2.0 ppm/s combined with a cooling rate at 3.0 and 1.0 °C/h kept 90.00% of the fish alive for 36.0 h (Table 2), which was better than when only hibernation was induced at a cooling rate of 1.0 °C/h (Table 1). Furthermore, CO_2_ aeration at 2.0 ppm/s combined with cooling at 3.0 °C/h only required 2.5 h to drop from 15.0 to 8.0 °C, which saved approximately threefold of the operating time than the only hypothermia hibernation method at a cooling rate of 1.0 °C/h. The recovered fish took an average of 18.5 ± 4.9 min to resume an upright equilibrium and swimming motion. When the fish were treated with CO_2_, the concentration of CO_2_ in their blood increased, causing changes in blood indicators, CO_2_ concentration in the blood, and their influence could last as long as 180 min despite the fish waking [12,14]. The cooling of live fish involved a process of temperature acclimation [25]; CO_2_ anesthesia could reduce the metabolism of the fish to compensate for the physiological changes due to stress caused by temperature changes. Therefore, the method combining CO_2_ anesthesia and hibernation could effectively prolong the survival time and rate and improve operation during the storage of large yellow croakers for food markets.

### 3.2. Blood Components, Glucose Levels, and Total Serum Protein of the Large Yellow Croaker Stored Using the Optimal Combined Method

The results of the blood analysis of large yellow croakers before and during hibernation and after recovery are shown in Table 3. Lymphocytes (LYM) were significantly elevated during hibernation and persistently increased after recovery. Red blood cells (RBC), hemoglobin (HGB), and hematocrit (HCT) increased during hibernation and decreased after recovery, but remained at higher levels than the basal values. The LYM and HCT are recognized as useful indicators for measuring stress response during anesthesia and cryopreservation. Their increase is synchronous with the rise in cortisol, which is primarily associated with metabolic shifts in accordance with escape responses [14,23]. The LYM and HCT results indicated that the large yellow croakers experienced stress during hibernation, which persisted after recovery. During hibernation, fish increased their oxygen-carrying capacity by releasing stored RBC and stimulating RBC production and HGB content in accordance with escape responses. The increase in RBC directly induced an elevation in HCT. The stress response did not disappear immediately after the fish recovered, consistent with the prior observations of strong stress responses in fish 30 min after recovery, with the subsequent return to basal values of stress response indicators, such as HCT and cortisol, within 90 min [14,26]. However, the changes in MCH before and during hibernation and after recovery were not significantly different, indicating that the combined method of hypothermia hibernation and CO_2_ anesthesia for the large yellow croaker did not influence the hemoglobin transport function.

**Figure 1:**
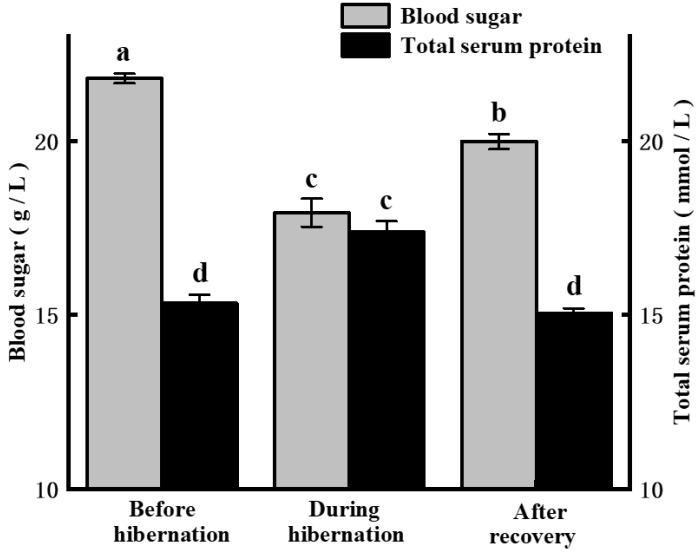
Blood sugar and total serum protein contents of the large yellow croaker before hibernation, during hibernation, and after recovery. Different letters indicate significant differences (*p* < 0.05).

The blood glucose content of hibernated large yellow croakers after hibernation (17.93 ± 0.37 g/L) was significantly (*p* < 0.05) lower than that of those before hibernation (21.79 ± 0.20 g/L) (Figure 1), and this increased rapidly to 19.98 ± 0.25 g/L after recovery. These changes were in accordance with those observed in crucian carp by Mi et al. [5]. In contrast, the total serum protein content of the hibernated large yellow croaker significantly increased (*p* < 0.05) from before hibernation to after hibernation (15.33 ± 0.17 mmol/L to 17.38 ± 0.27 mmol/L) and then immediately returned to normal levels (15.05 ± 0.21 mmol/L) when the fish recovered (Figure 1). Blood glucose is the main source of energy for life activities. The total serum protein reflects the metabolic intensity of liver function. The increases in the total serum protein and decrease in blood glucose during hibernation likely implied an increased metabolic intensity of liver function and decreased metabolic rate or activity of other organs in accordance with escape responses in fish [27]. Stress can rapidly lower the glycogen content in fish tissues that include muscle [28]. However, the regulation of energy metabolism may show the decreased expression of glycolytic genes and activation of other energy pathways during long-term storage under cold conditions. For example, the main energy-supplying metabolic pathway of glycolysis would shift to fat catabolism [29]. Additionally, Ylä-Ajos et al. [30] explained that prolonged hypothermia inhibits glycogen debranching enzymes and decreases glycogenolysis. These changes lead to a reduction in the amount of blood glucose and influence postmortem metabolism related to fishery product quality. However, the blood glucose level of recovered fish was significantly lower than that before hibernation (*p* < 0.05), suggesting that hibernation and accompanying stress responses were energy-demanding processes, and the fish could only hibernate for a limited time. Notably, the total serum protein levels were not significantly different before hibernation and after recovery (*p* > 0.05). Similarly, Mi et al. [5] reported that alkaline phosphatase and acid phosphatase activity, which are important enzymes in serum protein reflecting hepatocellular damage, significantly increased during hibernation, but immediately decreased to basal levels after the fish recovered. These results indicated that the influence of hibernation on the liver of the large yellow croaker could be quickly repaired within 36.0 h using this combinative method.

In this study, the optimized combined storage method was found to cause stress on the fish energy metabolism, resulting in changes in blood glucose, serum total protein, WBC, RBC, HGB, and HCT. However, these changes were mild and restorable, and were suitable for the long-term transportation of the larger yellow croaker.

### 3.3. Drip Loss

**Figure 2:**
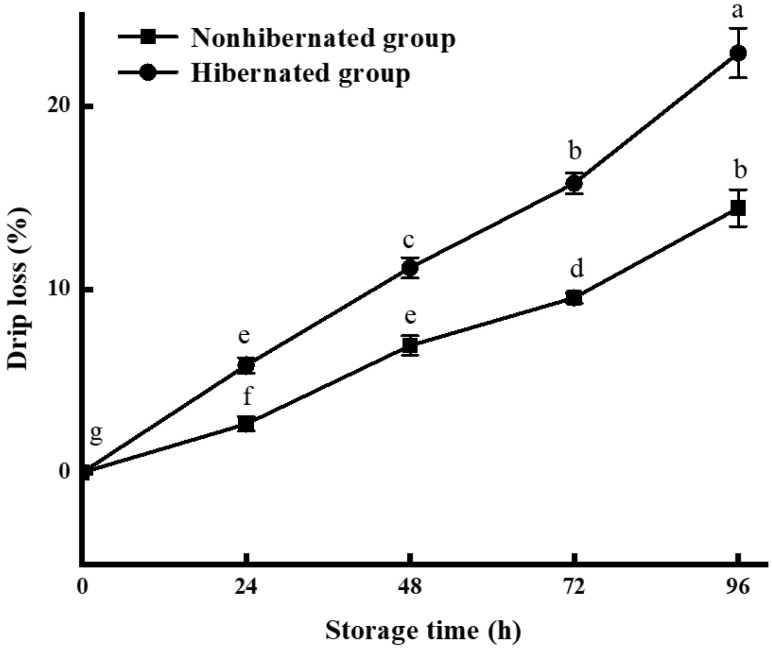
Drip loss of hibernated and nonhibernated large yellow croaker at 4 °C. Different letters indicate significant differences (*p* < 0.05).

The drip loss indicates the water-holding ability of muscle, especially the myofibril network, and is closely related to salt-soluble protein [31]. The drip loss of the hibernated large yellow croaker increased faster (*p* < 0.05) than that of the control group during storage (Figure 2). Our results indicated that the myofibrillar network in salt-soluble proteins of hibernated large yellow croakers was damaged during storage, primarily due to the hypoxia and stress response of the fish during hibernation and CO_2_ anesthesia, which caused the myofibril network to become fragile [32]. Bai et al. [7] showed that the water-holding capacity of channel catfish decreased after hibernation and gradually recovered to basal levels after domestication over 24 h at 20 °C. Therefore, the domestication of fish after recovery could minimize the effects of anesthesia and hibernation on fish quality.

### 3.4. pH and Lactic Acid

**Figure 3:**
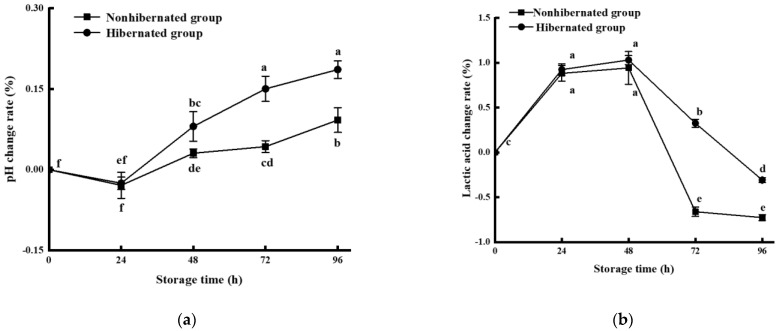
pH (**a**) and lactic acid (**b**) change rates of hibernated and nonhibernated large yellow croaker at 4 °C. Different letters indicate significant differences (*p* < 0.05).

During early storage, the decomposition of glycogen under hypoxic conditions leads to an increase in the lactic acid content and a decrease in pH [33,34]. As a result, proteins gradually decompose into alkaline substances, such as trimethylamine (TMA) and dimethylacetamide (DMA) under the action of enzymes and microorganisms, resulting in a net increase in pH [35,36]. As shown in Figure 3, the rate of changes in the pH in the hibernated control groups decreased within 24 h and then increased gradually with the extension of storage time. The increase in pH occurred at a faster rate in the hibernated group than the control group and was significantly different from the control after 48 h (*p* < 0.05). In contrast, the changes in lactic acid increased over 48 h and then declined rapidly as the storage time was extended. However, lactic acid in the control group decreased significantly faster than that in the hibernation group after 72 h (*p* < 0.05), mainly due to the accumulation of lactic acid in the live large yellow croaker caused by hypoxia during hibernation (the lactic acid contents in hibernated fish at 0 h were 10.74 ± 0.29 and 8.05 ± 0.89 in the controls (not shown in Figure 3b)). Changes in lactic acid and pH in the hibernation group were much slower and faster than those in the controls, meaning that the proteins in the hibernation group began decomposing earlier due to storage conditions. Thus, our results implied that the hibernation method affected the quality of the large yellow croaker during storage, but had no significant effect on changes in pH levels over 48 h and lactic acid over 72 h.

### 3.5. Color Variations

**Figure 4:**
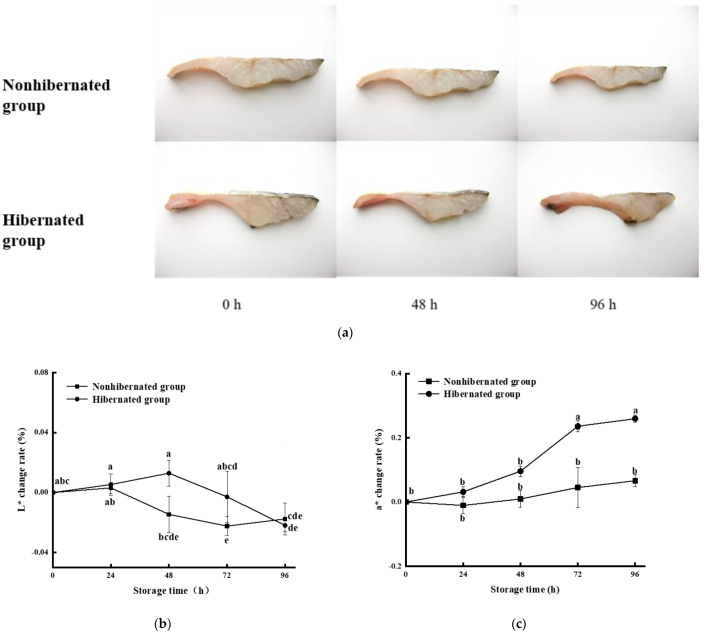
State change (**a**), and L* (**b**) and a* (**c**) change rates of hibernated and nonhibernated large yellow croaker at 4 °C. Different letters indicate significant differences (*p* < 0.05).

The differences in the fillet quality of the hibernated and control groups during storage are shown in Figure 4a. The change rate of the L* value was largest at 48 h (1.29%) and 24 h (0.31%) in the hibernated and control groups, respectively (Figure 4b). Meanwhile, the change rate of a* value of the hibernated group increased faster and was significantly different (4.53%) compared with that of the control (2.36%) after 72 h (Figure 4c). The main factor affecting color changes in the ordinary dorsal muscle (white muscle) of fish was the content and chemical state of hemoglobin [21,22]. During early storage, the hemoglobin reacted with oxygen to produce bright red oxyhemoglobin, causing increases in L* and a* values. Additionally, the hemoglobin of fish is particularly sensitive to changes in pH. Increases in pH promote the oxidation of fish muscle, resulting in the reddish-brown methemoglobin visible via the reddened and darkened muscle and leading to a continuous increase in the a* value and decrease in the L* value [37,38]. The rapid increase in the pH of hibernated fish (Figure 3a) mainly resulted in a rapid decrease in its L* value and a rapid increase in its a* value compared with the controls. In addition, increases in drip loss during early storage cause water exudation from the internal muscle and increase the water content on the muscle surface, resulting in an enhanced light reflection ability of muscle, namely, an increase in the L* value [39]. Thus, faster changes in the L* value of the hibernated group during early storage were mainly attributed to faster increases in drip loss (Figure 2).

In particular, the hibernation of fish led to a reduced respiration intensity and affected the binding ability of oxygen and hemoglobin to RBC, resulting in a high hemoglobin content and low oxyhemoglobin content [7,40]. The hibernated fish were killed on recovery (only 18.5 ± 4.9 min after fish were transferred to 15.0 °C seawater), and its fillet possibly had more hemoglobin that could combine with oxygen than the control group, which also led to rapid changes in color variations and likely prolonged increases in the L* value in the hibernated group.

### 3.6. Texture

**Figure 5:**
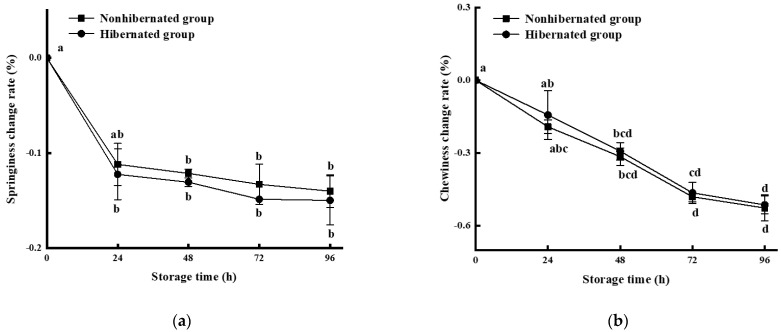
Springiness (**a**) and chewiness (**b**) change rates of hibernated and nonhibernated large yellow croaker at 4 °C. Different letters indicate significant differences (*p* < 0.05).

The rate of change in springiness in the hibernated and control groups dropped rapidly within 24 h (Figure 5a), then decreased slowly until the end of storage. Simultaneously, the rate of change in chewiness in the two groups gradually decreased with the storage time (Figure 5b). Springiness reflects the integrity and toughness of muscle fibers [41,42]; the springiness dropped during early storage due to the rigor of tissue and the breakdown of the Z line of myofibril, then decreased slowly with cell destruction caused by microorganisms and endogenous enzymes [43,44]. Chewiness reflects the energy consumption required for chewing food into a swallowable state, which is closely related to the overall stability of muscle tissue. That is, it is the result of the combined effects of hardness and intercellular cohesion [45,46]. The decrease in chewiness of the fish was due to the degradation of proteins and the decrease in the intercellular binding force under the action of enzymes, microorganisms, and metabolites [47]. Although both the springiness and chewiness of the hibernated group decreased slightly faster than those of the control group, there were no significant differences between the two groups during storage (*p* > 0.05). Thus, this hibernation method had no significant influence on the rate of change in the physical properties of fish quality, such as the texture and structure of muscle during storage.

## 4. Conclusions

This study demonstrated that CO_2_ anesthesia combined with hibernation by rapid cooling (3 °C/h) could maintain 90.00% of large yellow croakers for 36.0 h, and was, therefore, an effective method of long-term storage for the fish during transportation through China. The results of the blood analysis indicated that the fish experienced stress under the combined storage method, which was mitigated after recovery; however, the hemoglobin transport function was not affected. The blood glucose content increased, while the total serum protein content decreased during hibernation, but they returned to the slightly lower basal levels on recovery, suggesting that most of the stress responses gradually disappeared. In other words, the combined storage method did not induce cellular damage in large yellow croakers. The drip loss rate of ordinary muscle of fish in the hibernation group was significantly different from that of the control group at 4.0 °C storage. However, there was no significant difference in the change rates of pH, lactic acid content, and color of the fish in the hibernated and control group within 24, 48, 24, and 48 h of storage, and the combined hibernation method did not affect the springiness and the chewiness. Nevertheless, the hibernation method did affect the quality of the fish as they were killed on recovery, and did not have enough time to repair the damage caused by hibernation stress. Fish quality parameters, including muscle color, determine the acceptability and price of fish. Thus, the fish would have to be sold or consumed immediately after recovery from hibernation, or on the appropriate domestication of hibernated fish after recovery from hibernation to experience the best quality, which needs to be taken into consideration.

## Figures and Tables

**Table 1:** Survival quantity and time of the large yellow croaker under CO_2_ anesthesia and hibernation.

CO_2_ anesthesia	**CO_2_ Aeration Rate (ppm/s)**	**Survival/Total (Survival Rate)**	**Survival Time (h)**
	2.0	10/11 (90.90%)	3.5
	7.0	7/12 (58.33%)	2.5
	12.0	4/11 (36.36%)	1.5
	17.0	1/10 (10.00%)	1.0
	22.0	0/13 (0.00%)	0.2
Hibernation	**Cooling Rate (** **°C/h)**	**Survival/Total (Survival Rate)**	**Survival Time (h)**
	1.0	9/10 (90.00%)	30.0
	3.0	6/11 (54.54%)	26.0
	5.0	3/9 (33.33%)	11.0
	7.0	1/12 (8.33%)	1.0
	9.0	0/12 (0.00%)	0.0

**Table 2:** Survival quantity and time of large yellow croaker under CO_2_ anesthesia combined with hibernation.

Hibernation combined with CO_2_ aeration rate of 2 ppm/s	**Cooling Rate (°C/h)**	**Survival/Total (Survival Rate)**	**Survival Time (h)**
	1.0	9/10 (90.00%)	36.0
	3.0	9/10 (90.00%)	36.0
	5.0	5/10 (50.00%)	15.0

**Table 3:** Blood routine of the large yellow croaker before hibernation, during hibernation, and after recovery.

Stage	LYM (10^9^/L)	RBC (10^12^/L)	HGB (g/L)	HCT (%)	MCV (fL)	MCH (pg)
Before hibernation	2.03 ± 0.11 ^c^	0.79 ± 0.02 ^b^	17.08 ± 0.76 ^b^	13.69 ± 0.10 ^b^	173.34 ± 0.23 ^a^	21.61 ± 0.52 ^a^
During hibernation	3.10 ± 0.04 ^b^	0.93 ± 0.04 ^a^	19.89 ± 0.16 ^a^	14.83 ± 0.19 ^a^	159.15 ± 0.50 ^b^	21.36 ± 1.09 ^a^
After recovery	3.97 ± 0.07 ^a^	0.87 ± 0.06 ^ab^	19.84 ± 0.44 ^a^	14.45 ± 0.33 ^a^	165.90 ± 0.73 ^ab^	21.87 ± 0.93 ^a^

LYM lymphocytes, RBC total red blood cell count, HCT hematocrit, HGB hemoglobin, MCV mean corpuscular volume, and MCH mean corpuscular hemoglobin. The values are expressed as mean ± SD. Different letters indicate significant differences (*p* < 0.05).

## Data Availability

The data that support the findings of this study are available within the article.

## References

[1] Zhao T.F., Benjakul S., Sanmartin C., Ying X.G., Ma L.K., Xiao Z.S., Yu J., Liu G.Q., Deng S.G. (2021). Changes of volatile flavor compounds in large yellow croaker (*Larimichthys crocea*) during Storage, as evaluated by headspace gas chromatography–ion mobility spectrometry and principal component analysis. Foods.

[2] Wu S.M., Chen J.R., Chang C.Y., Tseng Y., Pan B.S. (2020). Potential benefit of I-Tiao-Gung (*Glycine tomentella*) extract to enhance ornamental fish welfare during live transport. Aquaculture.

[3] Zhang Y.J., Wang W.S., Yan L., Glamuzina B., Zhang X. (2019). Development and evaluation of an intelligent traceability system for waterless live fish transportation. Food Control.

[4] Zhang Y., Fu Z., Xiao X., Zhang X., Li D. (2017). MW-MTM: A mobile wireless monitoring and traceability management system for water-free live transport of aquatic products. J. Food Process Eng..

[5] Mi H.B., Qian C.L., Mao L.C. (2012). Quality and biochemical properties of artificially hibernated crucian carp for waterless preservation. Fish Physiol. Biochem..

[6] Salin K.R. (2015). Live transportation of *Macrobrachium rosenbergii* (De Man) in chilled sawdust. Aquacult. Res..

[7] Bai C., Xiong G., Xu P., Li N., Wang J., Liao T. (2020). Effect of cold-anesthetization rate on blood biochemical parameters and muscle composition during live channel catfish *Ictalurus punctatus* waterless preservation. Fish Sci..

[8] Ross L.G., Sanchez B.J., Martinez P.C., Racotta I.S., Toledo Cuevas M. (2007). Anaesthesia, sedation and transportation of juvenile *Menidia estor* (Jordan) using benzocaine and hypothermia. Aquacult. Res..

[9] Pramod P.K., Ramachandran A., Sajeevan T.P., Thampy S., Pai S.S. (2010). Comparative efficacy of MS-222 and benzocaine as anaesthetics under simulated transport conditions of a tropical ornamental fish *Puntius filamentosus* (Valenciennes). Aquacult. Res..

[10] Kugino K., Tamaru S., Hisatomi Y., Sakaguchi T. (2016). Long-duration carbon dioxide anesthesia of fish using ultra fine (Nano-Scale) bubbles. PLoS ONE.

[11] Chalon J., Martin P., Roberts C., Ramanathan S., Katz R., Turndorf H. (1983). Anaesthetic Uptake by the goldfish: Effect of respiratory rate. Acta Anaesthesiol. Scand..

[12] Mitsuda H., Ueno S., Mizuno H., Ueda T., Fujikawa H., Nohara T., Fukada C. (1982). Levels of CO_2_ in arterial blood of carp under carbon dioxide anesthesia. J. Nutr. Sci. Vitaminol..

[13] Grttum J.A., Sigholt T. (1996). Acute toxicity of carbon dioxide on European seabass (*Dicentrarchus labrax*): Mortality and effects on plasma ions. Comp. Biochem. Physiol. A Physiol..

[14] Guan W.L., Zhao M.M., Liu T.T., Fan X., Chen D.W. (2017). Cooling combined with hyperoxic CO_2_ anesthesia is effective in improving the air exposure duration of tilapia. Sci. Rep..

[15] Roth B., Slinde E., Arildsen J. (2006). Pre or post mortem muscle activity in Atlantic salmon (*Salmo salar*). The effect on rigor mortis and the physical properties of flesh. Aquaculture.

[16] Marianna P., Di C.B., Philippe M., Huvet A., Quillien V., Milan M., Ferraresso S., Pegolo S., Patarnello T., Bargelloni L. (2018). Understanding the mechanisms involved in the high sensitivity of Pecten maximus larvae to aeration. Aquaculture.

[17] Zeng L., Ai C.X., Zheng J.L., Zhang J.S., Li W.C. (2019). Cu pre-exposure alters antioxidant defense and energy metabolism in large yellow croaker *Larimichthys crocea* in response to severe hypoxia. Sci. Total Environ..

[18] Gang J.H., Geewook S. (2010). Efficacy of benzocaine as an anaesthetic for Crucian carp (*Carassius carassius*). Vet. Anaesth. Analg..

[19] He H.J., Wu D., Sun D.W. (2014). Rapid and non-destructive determination of drip loss and pH distribution in farmed Atlantic salmon (*Salmo salar*) fillets using visible and near-infrared (Vis–Nir) hyperspectral imaging. Food Chem..

[20] Meyer-Rochow V.B., Devine C. (1986). Ultrastrucvtural observations and pH-measurements on red and white muscle from Antarctic fish. Polar Biol..

[21] Olsen S.H., Sorensen N.K., Stormo S.K., Elvevoll E.O. (2006). Effect of slaughter methods on blood spotting and residual blood in fillets of Atlantic salmon (*Salmo salar*). Aquaculture.

[22] Adeyemi K.D., Sabow A.B., Shittu R.M., Karim R., Karsani S.A., Sazili A.Q. (2016). Impact of chill storage on antioxidant status, lipid and protein oxidation, color, drip loss and fatty acids of semimembranosus muscle in goats. CyTA-J. Food.

[23] Jiang T., Yuan P., Hirasaka K., Hamada Y., Hara K., Tachibana K., Taniyama S. (2019). The effect of blood deposition on the degradation of the connective tissue of the yellowtail *Seriola quinqueradiata* during storage. Fish Sci..

[24] Lu D.L., Ma Q., Wang J., Li L.Y., Han S.L., Limbu S.M., Li D.L., Chen L.Q., Zhang M.L., Du Z.Y. (2019). Fasting enhances cold resistance in fish through stimulating lipid catabolism and autophagy. J. Physiol..

[25] Peng Z., Chen T., Shen J. (2014). Effects of cold acclimation and storage temperature on crucian carp (*Carassius auratus gibelio*) in a waterless preservation. Fish Physiol. Biochem..

[26] Sandblom E., Seth H., Sundh H., Sundell K., Axelsson M., Kiessling A. (2013). Stress responses in Arctic char (*Salvelinus alpinus* L.) during hyperoxic carbon dioxide immobilization relevant to aquaculture. Aquaculture.

[27] Kolanczyk R.C., Fitzsimmons P.N., McKim J.M., Erickson R.J., Schmieder P.K. (2003). Effects of anesthesia (tricaine methanesulfonate, MS222) on liver biotransformation in rainbow trout (*Oncorhynchus mykiss*). Aquatic. Toxicol..

[28] Haard N.F. (1992). Control of chemical composition and food quality attributes of cultured fish. Food Res. Int..

[29] Gracey A.Y., Fraser E.J., Li W.Z., Fang Y.X., Taylor R.R., Rogers J., Brass A., Cossins A.R. (2004). Coping with cold: An integrative, multitissue analysis of the transcriptome of a poikilothermic vertebrate. Proc. Natl. Acad. Sci. USA.

[30] Yl-Ajos M., Ruusunen M., Puolanne E. (2006). The significance of the activity of glycogen debranching enzyme in glycolysis in porcine and bovine muscles. Meat Sci..

[31] Lakshmanan R., Parkinson J.A., Piggott J.R. (2007). High-pressure processing and water-holding capacity of fresh and cold-smoked salmon (*Salmo salar*). LWT-Food Sci. Technol..

[32] Lin R., Cheng S.S., Wang S.Q., Tan M.Q., Zhu B.W. (2021). Influence of refrigerated storage on water status, protein oxidation, microstructure, and physicochemical qualities of Atlantic mackerel (*Scomber scombrus*). Foods.

[33] Li T., Hu W., Li J., Zhang X., Zhu J., Li X. (2012). Coating effects of tea polyphenol and rosemary extract combined with chitosan on the storage quality of large yellow croaker (*Pseudosciaena crocea*). Food Control.

[34] Eduardo E., Luís G., Jaime A. (2021). Effects of vacuum and modified atmosphere packaging on the quality and shelf-life of gray triggerfish (*Balistes capriscus*) fillets. Foods.

[35] Cai L., Wu X., Li X., Zhong K., Li Y., Li J. (2021). Effects of different freezing treatments on physicochemical responses and microbial characteristics of Japanese sea bass (*Lateolabrax japonicas*) fillets during refrigerated storage. LWT-Food Sci. Technol..

[36] Sungsri I.R., Benjakul S., Kijroongrojana K. (2011). Pink discoloration and quality changes of squid (*Loligo formosana*) during iced storage. LWT-Food Sci. Technol..

[37] Aranda R., He C., Worley C.E., Levin E.J., Li R., Olson J.S., Phillips G.N., Richards M.P. (2009). Structural analysis of fish versus mammalian hemoglobins: Effect of the heme pocket environment on autooxidation and hemin loss. Proteins.

[38] Jiang T., Miyazaki R., Hirasaka K., Yuan P.X., Yoshida A., Hara K., Tachibana K., Taniyama S. (2019). Effect of blood deposition phenomenon on flesh quality of yellowtail (*Seriola quinqueradiata*) during storage. J. Texture Stud..

[39] Wulf D.M., Wise J.W. (1999). Measuring muscle color on beef carcasses using the L*a*b* color space. J. Anim. Sci..

[40] Vijayan M.M., Leatherland J.F. (1998). Effect of stocking density on the growth and stress-response in brook charr, *Salvelinus fontinalis*. Aquaculture.

[41] Stien L.H., Hirmas E., Bjørnevik M., Karlsen O., Nortvedt R., Rora A.M.B., Sunde J., Kiessling A. (2010). The effects of stress and storage temperature on the colour and texture of pre-rigor filleted farmed cod (*Gadus morhua* L.). Aquacult. Res..

[42] Yang X.Y., Sebranek J.G., Luo X., Zhang W.G., Zhang M.M., Xu B.C., Zhang Y.M., Liang R.R. (2021). Effects of calcium salts on the physicochemical quality of cured beef sausages during manufacturing and storage: A potential calcium application for sausages with alginate casings. Foods.

[43] Taylor R.G., Fjaera S.O., Skjervold P.O. (2010). Salmon fillet texture is determined by myofiber-myofiber and myofiber-myocommata attachment. J. Food Sci..

[44] Hernández M.D., López M.B., Álvarez A., Ferrandini E., García García B., Garrido M.D. (2009). Sensory, physical, chemical and microbiological changes in aquacultured meagre (*Argyrosomus regius*) fillets during ice storage. Food Chem..

[45] Johnston I.A., Li X., Vieira V., Nickell D., Dingwall A., Alderson R., Campbell P., Bickerdike R. (2006). Muscle and flesh quality traits in wild and farmed Atlantic salmon. Aquaculture.

[46] Stoller G.M., Zerby H.N., Moeller S.J., Baas T.J., Johnson C., Watkins L.E. (2003). The effect of feeding ractopamine (Paylean) on muscle quality and sensory characteristics in three diverse genetic lines of swine. J. Anim. Sci..

[47] Riebroy S., Benjakul S., Visessanguan W., Tanaka M. (2007). Effect of iced storage of bigeye snapper (*Priacanthus tayenus*) on the chemical composition, properties and acceptability of Som-fug, a fermented Thai fish mince. Food Chem..

